# The diagnostic utility of gram stain microscopy in paediatric septic arthritis – a retrospective case study

**DOI:** 10.1186/1753-6561-6-S4-P35

**Published:** 2012-07-09

**Authors:** J Wong, R Barksfield, R Hutchinson

**Affiliations:** 1 Norwich Medical School, University of East Anglia and Norfolk and Norwich University Hospital, UK

## Introduction

The diagnosis of septic arthritis in children remains challenging despite reasonable evidence for the use of laboratory tests in diagnosis. There is also limited data on the diagnostic utility of gram stain microscopy in diagnosis. We therefore aim to establish the diagnostic utility of gram stain and predictive clinical and laboratory features of paediatric septic arthritis.

## Methods

We conducted a retrospective review of all patients of 16 years and under that underwent aspiration with or without washout of suspected septic joint from January 2005 to March 2011. Cases were defined as any patient with an organism identified on microbiology culture. The association between clinical features, laboratory results, operative findings and gram stain examination were compared against final culture results with chi-square test (for categorical data) and Mann Whitney test (for non-parametric data).

## Results

Twenty three paediatric patients were identified during the time period, of which 9 (39%) had positive culture. There was no statistically significant data to show that clinical features or operative findings were predictive of final results. Of the blood test results found, CRP has statistical significant rise (p=0.01) in culture positive (mean 33, IQR 8-293) septic joints compared to culture negative (mean 107, IQR 65-190) with CRP >65 rendering sensitivity 100% and specificity 78%. Gram stain microscopy showed 33.3% sensitivity and 100% specificity.

## Conclusions

Presenting features, operative findings and most laboratory tests are unhelpful in predicting diagnosis for septic arthritis. However a high CRP (>65) may be useful diagnostic tool proven by high sensitivity. Positive gram stain is strongly predictive of culture positive septic arthritis although diagnosis cannot be excluded on the basis of negative gram stain. Further research should be conducted using CRP and gram stain alongside each other as diagnostic utility for paediatric septic arthritis as demonstrated by the research.

